# 13-hydroxy linoleic acid increases expression of the cholesterol transporters ABCA1, ABCG1 and SR-BI and stimulates apoA-I-dependent cholesterol efflux in RAW264.7 macrophages

**DOI:** 10.1186/1476-511X-10-222

**Published:** 2011-11-30

**Authors:** Ines Kämmerer, Robert Ringseis, Ronald Biemann, Gaiping Wen, Klaus Eder

**Affiliations:** 1 Institute of Animal Nutrition and Nutrition Physiology, Justus-Liebig-University Giessen, Heinrich-Buff-Ring 26-32, 35390 Giessen, Germany

**Keywords:** Peroxisome proliferator-activated receptors, Cholesterol efflux, Macrophage, Oxidized fatty acids

## Abstract

**Background:**

Synthetic activators of peroxisome proliferator-activated receptors (PPARs) stimulate cholesterol removal from macrophages through PPAR-dependent up-regulation of liver × receptor α (LXRα) and subsequent induction of cholesterol exporters such as ATP-binding cassette transporter A1 (ABCA1) and scavenger receptor class B type 1 (SR-BI). The present study aimed to test the hypothesis that the hydroxylated derivative of linoleic acid (LA), 13-HODE, which is a natural PPAR agonist, has similar effects in RAW264.7 macrophages.

**Methods:**

RAW264.7 macrophages were treated without (control) or with LA or 13-HODE in the presence and absence of PPARα or PPARγ antagonists and determined protein levels of LXRα, ABCA1, ABCG1, SR-BI, PPARα and PPARγ and apolipoprotein A-I mediated lipid efflux.

**Results:**

Treatment of RAW264.7 cells with 13-HODE increased PPAR-transactivation activity and protein concentrations of LXRα, ABCA1, ABCG1 and SR-BI when compared to control treatment (P < 0.05). In addition, 13-HODE enhanced cholesterol concentration in the medium but decreased cellular cholesterol concentration during incubation of cells with the extracellular lipid acceptor apolipoprotein A-I (P < 0.05). Pre-treatment of cells with a selective PPARα or PPARγ antagonist completely abolished the effects of 13-HODE on cholesterol efflux and protein levels of genes investigated. In contrast to 13-HODE, LA had no effect on either of these parameters compared to control cells.

**Conclusion:**

13-HODE induces cholesterol efflux from macrophages via the PPAR-LXRα-ABCA1/SR-BI-pathway.

## Background

Although dietary consumption of oxidized fats (OF) is known to cause some unfavourable effects (e.g., oxidative stress, depletion of antioxidants; [1-3]), experiments in laboratory animals and pigs consistently demonstrated that administration of OF reduces lipid concentrations (triacylglycerols and cholesterol) in liver and plasma (reviewed in [4]). Recent evidence suggests that activation of the peroxisome proliferator-activated receptor α (PPARα) pathway in the liver is largely responsible for the lipid lowering action of OF [5-7]. PPARα is a ligand-activated transcription factor which controls a comprehensive set of genes involved in most aspects of lipid catabolism [8,9]. Thus, targeting PPARα by the administration of pharmacological PPARα activators, e.g., fenofibrate, bezafibrate, gemfibrozil, is an effective approach for the treatment of hyperlipidemia [10].

Besides targeting lipid catabolism in the liver and regulating plasma lipid concentrations, synthetic PPARα activators also directly influence vascular function in a beneficial manner through negatively regulating the expression of pro-inflammatory genes in vascular cells such as endothelial cells, smooth muscle cells, and macrophages and inducing genes involved in macrophage cholesterol homeostasis [11-13]. These direct atheroprotective together with the lipid lowering effects are largely responsible for the observation that pharmacological PPARα activators cause an inhibition of atherosclerosis development [14-17]. Interestingly, in a recent study it could be demonstrated that dietary administration of an OF also causes activation of PPARα in the vasculature, inhibits expression of pro-inflammatory vascular adhesion molecules, whose expression is negatively regulated by PPARα, and inhibits atherosclerotic plaque development in the low-density lipoprotein receptor deficient mouse model of atherosclerosis [18]. These findings suggest that OF exerts similar effects as pharmacological PPARα agonists.

The components of OF which are supposed to be responsible for PPARα activation are hydroxy and hydroperoxy fatty acids, such as 13-hydroxy octadecadienoic acid (13-HODE) or 13-hydroperoxy octadecadienoic acid (13-HPODE). These substances are formed during oxidation of dietary lipids and absorbed from the intestine following ingestion of these fats [19,20]. Using different experimental approaches, such as ligand binding studies, transactivation assays and cell culture experiments, it was shown that these oxidized fatty acids are potent ligands and activators of PPARα [21-24]. An animal experiment revealed that feeding a diet supplemented with 13-HPODE reduces plasma triacylglycerol concentrations indicating that oxidized fatty acids are indeed the mediators of the lipid lowering effects of OF [25]. Whether oxidized fatty acids are also responsible for the observation that OF modulates the expression of PPAR-dependent genes in the vasculature [18], has not been studied yet. Therefore, the present study aimed to test the hypothesis that the hydroxylated derivative of linoleic acid, 13-HODE, induces genes involved in macrophage cholesterol homeostasis, such as liver × receptor α (LXRα), ATP-binding cassette transporter A1 (ABCA1), ABCG1 and scavenger receptor class B type 1 (SR-BI), and increases cholesterol removal from macrophages in a PPAR-dependent manner. Recent studies showed that synthetic activators of PPARα stimulate cholesterol removal from macrophages, an important step in reverse cholesterol transport, through PPAR-dependent up-regulation of LXRα [26-28], which serves as an intracellular cholesterol sensor and positively regulates expression of cholesterol exporters such as ABCA1, ABCG1 and SR-BI [29].

## Materials and methods

### Cell culture and treatments

Mouse RAW264.7 cells, obtained from LGC Promochem (Wesel, Germany), were grown in DMEM medium (Gibco/Invitrogen, Karlsruhe, Germany) supplemented with 10% fetal calf serum, 4 mmol/L L-glutamine, 4.5 g/L glucose, 1 mmol/L sodium pyruvate, 1.5 g/L sodium bicarbonate and 0.5% gentamycin. Cells were maintained at 37°C in a humidified atmosphere of 95% air and 5% CO_2_. RAW264.7 cells were plated in 6-well plates at a density of 1 × 10^6^/well for western blot analysis and at a density of 8 × 10^5^/well for cholesterol analysis. After reaching 80% confluence, cells were treated with LA (≥96% pure) and 13-HODE (≥96% pure; both from Sigma-Aldrich, Taufkirchen, Germany) at the concentrations indicated for 24 h. Cells treated with vehicle alone (ethanol) were used as controls. Incubation media containing fatty acids were prepared by diluting the fatty acid stock solutions (100 mmol/L LA and 2.5 mmol/L 13-HODE in ethanol) with DMEM medium to 100 μmol/L (LA) and 2.5 μmol/L (13-HODE), as also described from others [30]. After addition of the fatty acids to the medium, the medium was gently vortexed at RT to ensure complete solubility of the added fatty acids. No signs of precipitation could be observed. Due to the presence of BSA in the medium, it is expected that most of the added fatty acids was bound to albumin which serves as the natural delivery molecule for free fatty acids in plasma. The concentration of 13-HODE used was based on the knowledge that this fatty acid can be found in human blood in the low μmolar range [31]. Incubation media of control cells contained the same vehicle (ethanol) concentration of 0.1% (v/v). Specific precautions other than appropriate storage conditions (-20°C, in the dark) were not taken to prevent oxidation of LA and 13-HODE. 13-HODE has been reported to be very stable against oxidation as evidenced from air oxidation experiments with 13S-HODE which were carried out by addition of amounts of iron ions greatly surpassing the Fe^2+ ^concentration in biological samples [32]. Even under extreme conditions, such as elevated temperature (45-50°C) and enhanced reaction time (2 weeks), 95% of the 13S-HODE was recovered unchanged by GC-MS analysis [32]. For experiments using PPAR inhibitors, cells were pre-treated with either 10 μmol/L of the PPARα selective antagonist GW6471 (Sigma-Aldrich) or 20 μmol/L of the PPARγ selective antagonist GW9662 (Sigma-Aldrich) 4 h before treatment with fatty acids. All experiments were performed between passages 5 and 8.

### Western blot analysis

After treatment of cells as indicated above, cells were lysed with RIPA lysis buffer (50 mmol/L Tris pH 7.5, 150 mmol/L NaCl, 1 mmol/L EDTA, 1% Triton X-100, 1% sodium deoxycholate, 0.1% SDS) containing protease inhibitor cocktail (Sigma), and protein concentrations of lysates determined by the BCA assay (VWR, Darmstadt, Germany). Equal amounts of protein were electrophoresed by 7.5% SDS-PAGE for ABCA1 and ABCG1 and 10% SDS-PAGE for SR-BI and LXRα and transferred to a nitrocellulose membrane. The membranes were blocked at 4°C in blocking solution (5% skim milk in Tris buffered saline with Tween-20 [TBS-T]: 50 mmol/L Tris, 150 mmol/L NaCl, pH 7.5, 0.2% Tween-20), and then incubated with primary antibodies against ABCA1 (1:1,000, Novus Biologicals), ABCG1 (1:2000, Abcam), β-Actin (1:1,000, Novus Biologicals), SR-B1 (1:1,000, Novus Biologicals), LXRα (1:500, Affinity BioReagents) for 2 h at room temperature or overnight at 4°C depending on the antibody used. The membranes were washed with TBS-T, and incubated with a horseradish peroxidase conjugated secondary anti-mouse IgG antibody (1:10,000, Jackson Immuno Research) or anti-rabbit IgG antibody (1:10,000, Sigma-Aldrich) for 1.5 h at room temperature. Afterwards blots were developed using ECL Advance (GE Healthcare Europe, Freiburg, Germany) for polyclonal antibodies and ECL Plus (GE Healthcare Europe) for monoclonal antibodies. The signal intensities of specific bands were detected with Bio-Imaging system (Syngene, Cambridge, UK) and quantified using Syngene GeneTools software (Nonlinear Dynamics, USA).

### Analysis of cholesterol content in medium and cells

After pre-treatment with or without PPAR antagonists and treatment of macrophage cells with or without fatty acids as indicated above, cells were incubated again with the antagonists for 4 h and afterwards with or without the corresponding fatty acids in the presence or absence of apolipoprotein A-I (apoA-I) (30 μg/mL) for 24 h. Afterwards, medium was collected and removed from detached cells by a centrifugation step, and the cell monolayer washed twice with PBS. Cellular lipids were extracted with a mixture of hexane and isopropanol (3:2, v/v) and lipids in the medium were extracted with a mixture of chloroform and methanol (2:1, v/v). Lipid extracts were dried under a stream of nitrogen and total cholesterol concentrations were determined using an enzymatic assay from Biocon (Vöhl-Marienhagen, Germany). Cholesterol concentrations were related to cellular protein content as determined by the BCA protein assay kit.

### Transient transfection and dual luciferase assay

RAW264.7 cells were plated in 24-well plates at a density of 5 × 10^5^/well. After reaching 70% confluence, cells were transiently transfected with 500 ng of a 3 × ACO-PPRE reporter vector (containing three copies of consensus PPRE from the ACO promoter in front of a luciferase reporter gene; a generous gift from Dr. Sander Kersten, Nutrigenomics Consortium, Top Institue (TI) Food and Nutrition, Wageningen, Netherlands) using FuGENE 6 transfection reagent (Roche Diagnostics, Mannheim, Germany) according to the manufacturer's protocol. Cells were also co-transfected with 50 ng of pGL4.74 Renilla luciferase (encoding the renilla luciferase reporter gene; Promega, Mannheim, Germany), which was used as an internal control reporter vector to normalize for differences in transfection efficiency. Following transfection, cells were treated with either WY-14,643 (as positive control), LA, 13-HODE or vehicle only (DMSO and ethanol) at the concentrations indicated for 24 h. Afterwards, cells were washed with PBS and lysed with passive lysis buffer (Promega). Luciferase activities were determined with the Firefly and Renilla Luciferase Assays (PJK, Kleinblittersdorf, Germany) according to the manufacturer's instructions using a Mithras LB940 luminometer (Berthold Technologies, Bad Wildbad, Germany) as described recently in more detail [33].

### Statistical analysis

Data were subjected to either Student's t-test or one-way ANOVA using the Minitab Statistical Software Rel. 13.0 (Minitab, State College, PA, USA). For statistically significant F values, individual means of the treatment groups were compared by Fisher's multiple range test. Means were considered significantly different for P < 0.05.

## Results

### Effects of 13-HODE and LA on PPAR transactivation activity and PPAR protein levels in RAW264.7 macrophages

**Figure 1:**
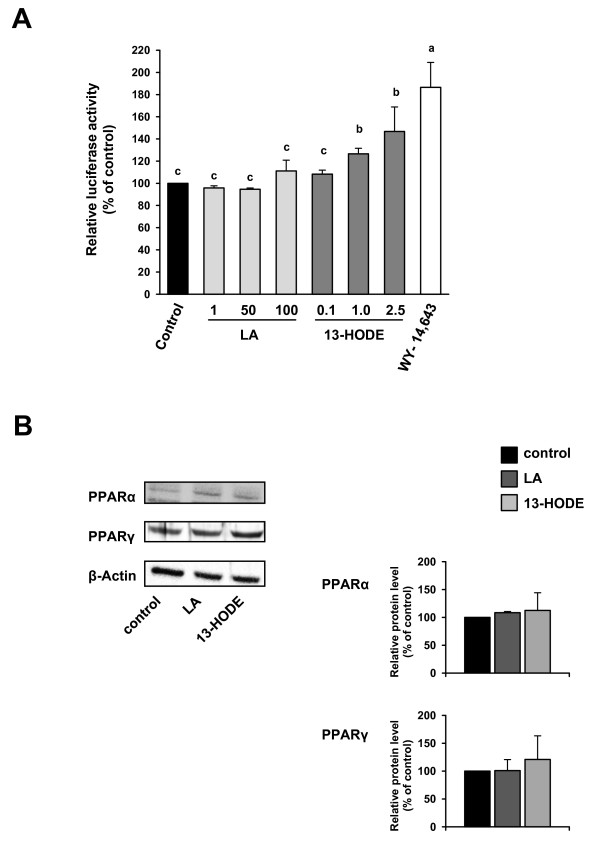
**Effects of 13-HODE, LA and WY-14,643 on PPAR/PPRE transactivation activity and PPAR protein levels in RAW264.6 macrophages**. A, RAW264.7 cells were transiently transfected with 3 × ACO-PPRE reporter vector. After transfection, cells were treated or not with 0.1-2.5 μmol/L 13-HODE, 1-100 μmol/L LA and 50 μmol/L WY-14,643 for 24 h. Afterwards, cells were lysed, and luciferase activities of the ACO-PPRE firefly luciferase vector and a co-transfected renilla luciferase vector determined by a dual luciferase assay. Bars represent means ± SD from four independent experiments (n = 4). Data are expressed as percentage of relative luciferase activity of vehicle control cells. Results from statistical analysis are indicated: Significant effects are denoted with an asterisk (*P *< 0.05). B, RAW264.7 cells were treated with 2.5 μmol/L 13-HODE, 100 μmol/L LA or vehicle (ethanol) for 24 h. Afterwards, cells were lysed and subsequently processed for western blotting as described in the materials and methods section. Representative immunoblots specific for PPARα, PPARγ, and β-actin which was used for normalization are shown. Bars represent data from densitometric analysis and are means ± SD from three independent experiments (n = 3). Data are expressed as percentage of protein concentration of vehicle control cells.

To study the effect of 13-HODE and LA on the activation of the PPAR signalling pathway in macrophages, RAW264.7 were transiently transfected with a reporter plasmid containing 3 copies of the consensus PPRE in front of a luciferase reporter and studied the stimulation of the reporter activity by 13-HODE and LA as well as by the synthetic PPARα agonist WY-14,643. Treatment with WY-14,643 as a positive control increased PPAR-responsive reporter activity by about 90% compared to treatment with vehicle alone (*P *< 0.05; Figure 1A). Treatment with 13-HODE dose-dependently increased the PPAR-responsive reporter activity compared to treatment with vehicle alone (*P *< 0.05; Figure 1A); incubating RAW264.7 cells with 1.0 and 2.5 μmol/L of 13-HODE increased the PPAR-responsive reporter activity by about 28 and 50%, respectively, compared to vehicle control. Incubation of macrophages with increasing concentrations of LA had no effect on the PPAR-responsive reporter activity when compared to macrophages treated with vehicle alone (Figure 1A); there was only a numerical, but not significant increase in the PPAR-responsive reporter activity at the highest concentration of LA (100 μmol/L) when compared to vehicle control. Protein concentrations of PPARα and PPARγ did not differ between control macrophages and macrophages treated with either 2.5 μmol/L 13-HODE or 100 μmol/L LA (Figure 1B).

### Effects of 13-HODE and LA in the presence and absence of PPARα and PPARγ selective antagonists on relative protein concentrations of ABCA1, ABCG1, SR-BI and LXRα in RAW264.7 macrophages

**Figure 2:**
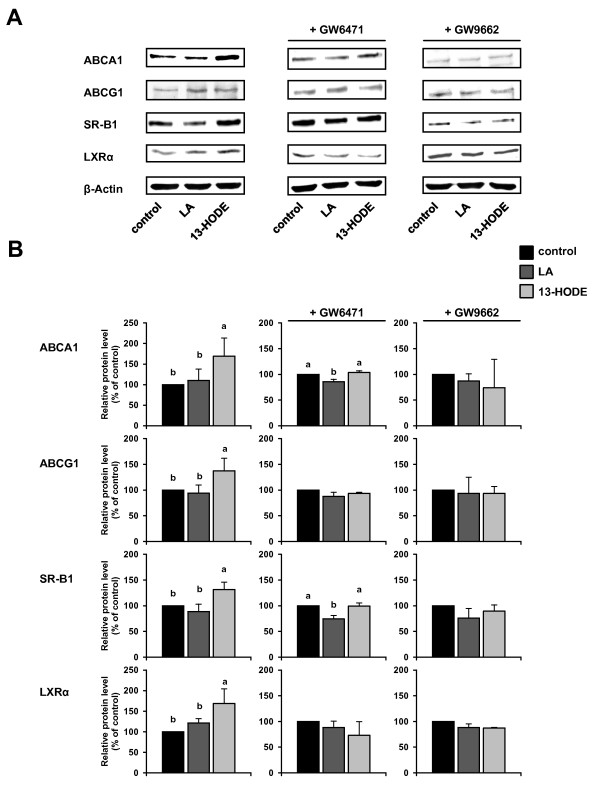
**Effects of 13-HODE and LA in the presence and absence of PPARα and PPARγ selective antagonists on molecular markers of cholesterol homeostasis in RAW264.7 macrophages**. RAW264.7 cells were pre-treated without or with the PPARα selective antagonist GW6471 or the PPARγ selective antagonist GW9662 and subsequently treated without (vehicle control) or with 2.5 μmol/L 13-HODE or 100 μmol/L LA for 24 h. Afterwards, cells were lysed and subsequently processed for western blotting as described in the materials and methods section. A, Representative immunoblots specific for ABCA1, ABCG1, SR-BI, LXRα, and β-actin which was used for normalization are shown. B, Bars represent data from densitometric analysis and are means ± SD from three independent experiments (n = 3). Data are expressed as percentage of protein concentration of vehicle control cells. Results from statistical analysis are indicated: Significant effects are denoted with superscript letters. Bars marked without a common superscript letter significantly differ (*P *< 0.05).

**Figure 3:**
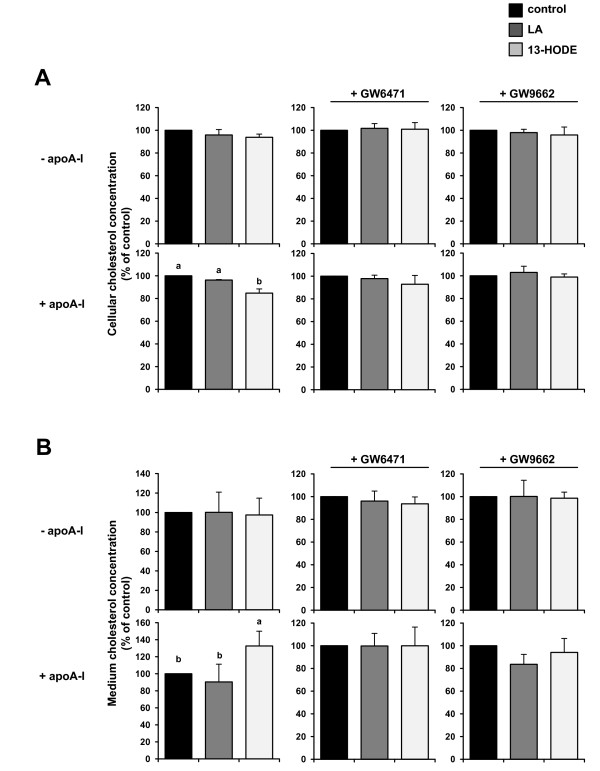
**Effects of 13-HODE and LA on cholesterol concentrations in cells and medium of macrophages in macrophages in the presence and absence of apoA-I and PPARα and PPARγ antagonists**. After pre-treatment without or with PPAR antagonists for 4 h and treatment of RAW264.7 macrophage cells without (vehicle control) or with 2.5 μmol/L 13-HODE or 100 μmol/L LA for 20 h, cells were incubated again without or with the antagonists for 4 h and afterwards without or with the corresponding fatty acids in the presence or absence of apolipoprotein A-I (apoA-I) (30 μg/mL) for 24 h. Afterwards, medium was collected, and cells were washed with PBS. Total lipids were extracted from medium and cells and concentrations of cholesterol determined as described in the materials and methods section. A, Cellular and B, medium cholesterol concentrations were related to cellular protein content. Bars represent means ± SD from four independent experiments (n = 4). Data are expressed as percentage of cholesterol concentration in cells and medium of control cells. Results from statistical analysis are indicated: Significant effects are denoted with an asterisk (*P *< 0.05).

To explore the involvement of PPARα and PPARγ in the action of 13-HODE on proteins regulating cholesterol homeostasis, cells were pre-treated without or with selective PPARα and PPARγ antagonists prior to treatment with fatty acids. In the absence of an antagonist, 2.5 μmol/L of 13-HODE increased protein levels of ABCA1, ABCG1, SR-BI and LXRα in RAW264.7 macrophages (*P *< 0.05; Figure 2A and 2B), whereas 100 μmol/L of LA had no effect (Figure 3A and 3B). When cells were pre-treated with either the PPARα antagonist GW6471 or the PPARγ antagonist GW9662 the effect of 13-HODE on the concentrations of these proteins was completely abolished (Figure 2A and 2B). In cells treated with LA, the pre-treatment with GW6471 caused a 15-25% decrease in the protein levels of ABCA1 and SR-BI (*P *< 0.05; Figure 2A and 2B), whereas protein levels of ABCG1 and LXRα remained unaffected. Pre-treatment with GW9662 did not alter the effect of LA on protein levels of ABCA1, ABCG1, SR-BI and LXRα in comparison to treatment without PPARα or PPARγ antagonist (Figure 2A and 2B).

### Effects of 13-HODE and LA on cholesterol concentrations in macrophages in the presence and absence of apoA-I and PPARα and PPARγ antagonists

To investigate whether the 13-HODE-induced alterations of the expression of proteins involved in cholesterol homeostasis had an effect on macrophage cholesterol content, we determined the cholesterol concentrations of cells and medium after treatment with 13-HODE and LA, both in the presence and absence of the extracellular lipid acceptor apo-AI. In the absence of apoA-I, cholesterol concentrations in cells and medium did not differ between control macrophages and macrophages treated with either LA or 13-HODE (Figure 3A and 3B). In the presence of apoA-I, treatment with 13-HODE decreased cellular cholesterol concentration by approximately 15% (*P *< 0.05; Figure 3A and 3B) and increased cholesterol concentration in medium by approximately 25% when compared to treatment with vehicle alone (*P *< 0.05; Figure 3A and 3B). In contrast, treatment with LA in the presence of apoA-I had no effect on cholesterol concentrations in cells and medium when compared to control treatment (Figure 3A and 3B). When cells were pre-treated with either the PPARα antagonist GW6471 or the PPARγ antagonist GW9662 the effect of 13-HODE on cellular and medium cholesterol concentration was completely abolished (Figure 3A and 3B).

## Discussion

Pharmacological PPAR ligands have been demonstrated to induce cholesterol removal from macrophages and to prevent macrophage foam cell formation through alterations in the expression of genes critically involved in macrophage cholesterol homeostasis [26-28]. Feeding OF was repeatedly shown to cause PPARα activation in tissues of different species [4]. This effect has been attributed to characteristic substances of OF such as hydroxylated fatty acids, e.g. 13-HODE, which are known ligands of PPARs [34,35]. The present study shows that 13-HODE moderately, but significantly lowers the cellular cholesterol content of macrophages while increasing the cholesterol content in the medium when apo-AI, the main apo of high density lipoprotein (HDL) particles, is present in the culture medium as an extracellular cholesterol acceptor. The export of cholesterol to acceptors such as apoA-I or HDL is an important part of the reverse cholesterol transport responsible for redistribution of cholesterol from peripheral tissues to the liver. Recent studies in RAW264.7 macrophages provided evidence that apoA-I is internalized by endocytosis into the macrophage where it acquires free cholesterol from intracellular pools before it is resecreted by exocytosis (novel model of cholesterol efflux called retroendocytosis), and that apoA-I internalization is required for transporter-mediated cholesterol efflux [36]. In the absence of apoA-I, no effect of 13-HODE on macrophage cholesterol content and cholesterol content in the incubation medium was observed. Thus, our findings indicate that 13-HODE stimulates specifically apoA-I-dependent cholesterol efflux in macrophages, an effect that is also known from synthetic PPAR ligands [26-28]. Interestingly, a previous study has shown that dietary oxidized fatty acids enhance intestinal cell apoA-I production via a PPAR-dependent process [37]. Although it has to be considered that plasma HDL levels are also determined by hepatic apoA-I synthesis and nascent HDL particle secretion, these previous findings together with our findings herein may be indicative of the ability of oxidized fatty acids to stimulate reverse cholesterol transport. Interestingly, evidence from feeding studies indeed shows that treatment of rats and guinea pigs with oxidized fat increases HDL cholesterol concentrations in plasma [3,38]. In pigs, however, which are better model objects for humans, no effect of oxidized fat on HDL cholesterol concentrations apoA-I production was found [39]. Epidemiological associations between oxidized fat intake and plasma HDL cholesterol in humans have not been established. This is probably explained by the fact that it is difficult to estimate the intake of oxidized fat.

On the molecular level, reduction of macrophage cholesterol accumulation and stimulation of cholesterol efflux from macrophages to extracellular lipid acceptors by PPAR agonists has been explained by an up-regulation of LXRα and subsequent induction of macrophage cholesterol exporters [26-28], like ABCA1 and ABCG1, which are direct LXRα target genes. Induction of SR-BI, which facilitates a bidirectional flux of free cholesterol between cells and lipoproteins, in response to PPAR agonists [40,41] is also considered to contribute to the increased macrophage cholesterol efflux and reverse cholesterol transport. Like ABCA1 and ABCG1, SR-BI promoter activity and protein levels are also positively regulated by LXRα through a functional LXR response element in its gene promoter [42]. Up-regulation of LXRα in response to PPAR agonists is attributed to the fact that LXRα is regulated by PPARs through a functional PPRE in the LXRα gene promoter [28,43]. Given that the blockade of PPARα or PPARγ by the use of selective PPARα or PPARγ antagonists in RAW264.7 cells resulted in a complete loss of the stimulatory effect of 13-HODE on LXRα, ABCA1, ABCG1 and SR-BI and cholesterol efflux, we suggest that 13-HODE exerted its effect on macrophage cholesterol homeostasis in a PPAR ligand-like manner. Conversely, the lack of effect of LA on cellular and medium cholesterol content and expression of LXRα, ABCA1, ABCG1 and SR-BI is probably explained by its failure to cause PPAR activation in RAW264.7 macrophages. The failure of LA to cause PPAR activation is likely due to the lower binding affinity of PPARs for unoxidized fatty acids compared with oxidized fatty acids like 13-HODE [44]. In line with this assumption are observations from several independent groups showing that LA does not induce PPAR target genes in both murine RAW264.7 [45,46] and human THP-1 macrophages [47].

As regards our observations with LA, it has to be mentioned that some studies reported that LA even decreases protein levels of ABCA1 and/or ABCG1 in either J774 macrophages or RAW264.7 macrophages [48-51]. Although it is difficult to provide a definite reason for this discrepancy, it is well known from the literature that cell culture studies dealing with fatty acids, in particular with LA, provided very controversial results [52]. Important reasons that may be responsible for these discrepancies could be differences in the passage number of cells or differences in the treatment regime, such as time of exposure and fatty acid concentration. Regarding the latter point, it is worth mentioning that in two of the abovementioned studies [48,49] the concentration of LA in the medium was higher than in the present study.

Recent studies demonstrated that PPAR activation also stimulates postlysosomal mobilization of cholesterol by induction of Niemann-Pick C (NPC)-1 and NPC-2 [53]. Both proteins control intracellular trafficking of cholesterol from the late endosomal compartment and lysosome, respectively, to the plasma membrane [54]. It has been suggested [53] that up-regulation of NPC-1 and -2 in response to PPAR agonists results in an enhanced availability of cholesterol at the cell membrane, and, thereby, contributes to increases in macrophage cholesterol efflux to extracellular acceptors and reverse cholesterol. For technical reasons we were not capable to determine protein expression of NPC-1 and NPC-2 in RAW264.7 macrophages. However, due to the observed similarities in the action of 13-HODE and synthetic PPAR ligands on macrophage cholesterol homeostasis we postulate that 13-HODE might also stimulate postlysosomal cholesterol mobilization. This has to be clarified in future studies. However, the regulation of cholesterol homeostasis in macrophages is complex and there are several other proteins important for maintenance of cholesterol homeostasis, including low density-lipoprotein (LDL) receptor, acyl-CoA cholesterol:acyltransferase, hydroxymethyl-glutaryl-CoA reductase, sterol regulatory element-binding proteins, steroidogenic acute regulatory (STAR)-related lipid transfer domain proteins, e.g. Star D4, and caveolin-1. Caveolin-1 for instance has been recently reported to be up-regulated by PPARα and PPARγ agonists [55]. It is therefore not unlikely that 13-HODE exerts its effect on macrophage cholesterol homeostasis also by altering the expression of one or more of these proteins. Thus, future studies applying transcriptomics or proteomics may be useful to get a more comprehensive insight into the mode of action of 13-HODE.

Oxidized fatty acids such as 13-HODE were also shown to activate the PPARγ isotype [34,35,56]. Although PPARγ is a less likely candidate for the mediation of the lipid lowering actions of OF, because PPARγ is poorly expressed in tissues with high rates of fatty acid catabolism like liver and skeletal muscle, it may be a putative mediator of the effect of 13-HODE on RAW264.7 macrophage cholesterol homeostasis. PPARγ is abundantly expressed in macrophage cell lines including RAW264.7 cells, as shown herein by western blotting, as well as primary macrophages [35]. In addition, synthetic PPARγ agonists were reported to stimulate macrophage cholesterol efflux by the same mechanisms as PPARα agonists, namely through activating the PPAR-LXR-pathway [12]. From our PPAR/PPRE-transactivation experiments, we cannot distinguish whether the activation of the reporter was due to activation of either PPARα or PPARγ because the PPRE from the mouse ACO promoter contained in the reporter plasmid used is known to be bound by both, PPARα and PPARγ [57]. Collectively, we suggest that the effects observed with 13-HODE on macrophage cholesterol homeostasis may be mediated by activating either PPARα, PPARγ or both of them.

Independent from the stimulatory effect of 13-HODE on proteins involved in macrophage cholesterol efflux, it is worth mentioning that with respect to 13-HODE also untoward effects have been reported in cell culture experiments, such as up-regulation of scavenger receptor CD36 which mediates the uptake of oxidized LDL [56]. Therefore, future studies using appropriate animal models of atherosclerosis, such as low density-lipoprotein-deficient or apolipoprotein E-deficient mice, have to clarify whether or not diets containing high levels of 13-HODE promote atherosclerosis development. Evidence from epidemiological studies concerning intake of oxidized fatty acids and cardiovascular disease risk is missing due to the lack of appropriate studies correlating the intake of oxidized fats with the incidence of cardiovascular diseases. Correlating the consumption of fried food with cardiovascular disease risk does not contribute to the clarification of this question because the lipid fraction of fried food contains not only oxidized fatty acids, but also large amounts of saturated fatty acids and trans-fatty acids which themselves influence cardiovascular disease risk.

## Conclusions

The present study shows that 13-HODE reduces cholesterol content in murine RAW264.7 macrophages and increases cholesterol content in the incubation medium probably by stimulating apoA-I-dependent cholesterol efflux in a PPAR-dependent manner. The 13-HODE-induced increase in cholesterol efflux from macrophages is likely due to PPAR-dependent up-regulation of LXRα and cholesterol transporters (ABCA1, ABCG1, SR-BI) which operate on cholesterol export to extracellular acceptors such as apoA-I/HDL. Because extensive accumulation of cholesterol by macrophages in the arterial wall leads to atherosclerosis, the present findings in macrophages suggest that the recently observed anti-atherogenic effects of OF [18] might be, at least in part, due to the inhibition of macrophage cholesterol accumulation and stimulation of reverse cholesterol transport caused by oxidized fatty acids such as 13-HODE. Future studies in human monocyte/macrophage cell lines, such as THP-1 cells, or human primary macrophages have to show whether the effects observed in murine macrophages also occur in human macrophages.

## Competing interests

The authors declare that they have no competing interests.

## Authors' contributions

IK carried out the experiments and participated in the interpretation of the results and the preparation of the manuscript. RR participated in the design of the study and in the interpretation of the results and prepared the manuscript. RB and GW carried out the experiments. KE conceived of the study and its design, coordinated work, participated in the interpretation of the results, and helped to draft the manuscript. All authors read and approved the final manuscript.

## References

[B1] Izaki Y Yoshikawa S Uchiyama M Effect of ingestion of thermally oxidized frying oil on peroxidative criteria in rats Lipids 1984 19 324 331 10.1007/BF02534782 6738310

[B2] Liu JF Huang CJ Tissue α-tocopherol retention in male rats is compromised by feeding diets containing oxidized frying oil J Nutr 1995 125 3071 3080 7500186 10.1093/jn/125.12.3071

[B3] Eder K Keller U Hirche F Brandsch C Thermally oxidized dietary fats increase the susceptibility of rat LDL to lipid peroxidation but not their uptake by macrophages J Nutr 2003 133 2830 2837 12949373 10.1093/jn/133.9.2830

[B4] Ringseis R Eder K Regulation of genes involved in lipid metabolism by dietary oxidized fat Mol Nutr Food Res 2011 55 109 121 10.1002/mnfr.201000424 21207516

[B5] Chao PM Chao CY Lin FJ Huang CJ Oxidized frying oil up-regulates hepatic acyl-CoA oxidase and cytochrome P450 4A1 genes in rats and activates PPARα J Nutr 2001 131 3166 3174 11739861 10.1093/jn/131.12.3166

[B6] Sülzle A Hirche F Eder K Thermally oxidized dietary fat upregulates the expression of target genes of PPARα in rat liver J Nutr 2004 134 1375 1383 15173399 10.1093/jn/134.6.1375

[B7] Ringseis R Muschick A Eder K Dietary Oxidized Fat Prevents ethanol-induced triacylglycerol accumulation and increases expression of PPARα target genes in rat liver J Nutr 2007 137 77 83 17182804 10.1093/jn/137.1.77

[B8] Mandard S Müller M Kersten S Peroxisome proliferator receptor α target genes Cell Mol Life Sci 2004 61 393 416 10.1007/s00018-003-3216-3 14999402 PMC11138883

[B9] Kersten S Seydoux J Peters JM Gonzalez FJ Desvergne B Wahli W Peroxisome proliferator-activated receptor α mediates the adaptive response to fasting J Clin Invest 1999 103 1489 1498 10.1172/JCI6223 10359558 PMC408372

[B10] Abourbih S Filion KB Joseph L Schiffrin EL Rinfret S Poirier P Pilote L Genest J Eisenberg MJ Effect of fibrates on lipid profiles and cardiovascular outcomes: a systematic review Am J Med 2009 122 962.e1 8 10.1016/j.amjmed.2009.03.030 19698935

[B11] Marx N Duez H Fruchart JC Staels B Peroxisome proliferator-activated receptors and atherogenesis: regulators of gene expression in vascular cells Circ Res 2004 94 1168 1178 10.1161/01.RES.0000127122.22685.0A 15142970

[B12] Chinetti G Lestavel S Bocher V Remaley AT Neve B Torra IP Teissier E Minnich A Jaye M Duverger N Brewer HB Fruchart JC Clavey V Staels B PPAR-α and PPAR-γ activators induce cholesterol removal from human macrophage foam cells through stimulation of the ABCA1 pathway Nat Med 2001 7 53 58 10.1038/83348 11135616

[B13] Gizard F Amant C Barbier O Bellosta S Robillard R Percevault F Sevestre H Krimpenfort P Corsini A Rochette J Glineur C Fruchart JC Torpier G Staels B PPARα inhibits vascular smooth muscle cell proliferation underlying intimal hyperplasia by inducing the tumor suppressor p16INK4a J Clin Invest 2005 115 3228 3238 10.1172/JCI22756 16239970 PMC1257531

[B14] Li AC Binder CJ Gutierrez A Brown KK Plotkin CR Pattison JW Valledor AF Davis RA Willson TM Witztum JL Palinski W Glass CK Differential inhibition of macrophage foam-cell formation and atherosclerosis in mice by PPARα, β/δ, and γ J Clin Invest 2004 114 1564 1576 15578089 10.1172/JCI18730 PMC529277

[B15] Hennuyer N Tailleux A Torpier G Mezdour H Fruchart JC Staels B Fiévet C PPARα, but not PPARγ, activators decrease macrophage-laden atherosclerotic lesions in a nondiabetic mouse model of mixed dyslipidemia Arterioscler Thromb Vasc Biol 2005 25 1897 1902 10.1161/01.ATV.0000175756.56818.ee 15994444

[B16] Ericsson CG Nilsson J Grip L Svane B Hamsten A Effect of bezafibrate treatment over five years on coronary plaques causing 20% to 50% diameter narrowing (the Bezafibrate Coronary Atherosclerosis Intervention Trial [BECAIT]) Am J Cardiol 1997 80 1125 1129 10.1016/S0002-9149(97)00626-7 9359536

[B17] Rubins HB Robins SJ Collins D Fye CL Anderson JW Elam MB Faas FH Linares E Schaefer EJ Schectman G Wilt TJ Wittes J Veterans Affairs High-Density Lipoprotein Cholesterol Intervention Trial Study Group. Gemfibrozil for the secondary prevention of coronary heart disease in men with low levels of high-density lipoprotein cholesterol N Engl J Med 1999 341 410 418 10.1056/NEJM199908053410604 10438259

[B18] Kämmerer I Ringseis R Eder K Feeding a thermally oxidised fat inhibits atherosclerotic plaque formation in the aortic root of LDL receptor-deficient mice Br J Nutr 2011 105 190 199 10.1017/S0007114510003478 20854700

[B19] Staprans I Rapp JH Pan XM Kim KY Feingold KR Oxidized lipids in the diet are a source of oxidized lipid in chylomicrons of human serum Arterioscler Thromb 1994 14 1900 1905 10.1161/01.ATV.14.12.1900 7981177

[B20] Staprans I Rapp JH Pan XM Feingold KR Oxidized lipids in the diet are incorporated by the liver into very low density lipoprotein in rats J Lipid Res 1996 37 420 430 9026539

[B21] König B Eder K Differential action of 13-HPODE on PPARα downstream genes in rat Fao and human HepG2 hepatoma cell lines J Nutr Biochem 2006 17 410 418 10.1016/j.jnutbio.2005.08.011 16216487

[B22] Mishra A Chaudhary A Sethi S Oxidized ω-3 fatty acids inhibit NF-κB activation via a PPARα-dependent pathway Arterioscl Thromb Vasc Biol 2004 24 1621 1627 10.1161/01.ATV.0000137191.02577.86 15231516

[B23] Muga SJ Thuillier P Pavone A Rundhaug JE Boeglin WE Jisaka M Brash AR Fischer SM 8S-lipoxygenase products activate peroxisome proliferator-activated receptor α and induce differentiation in murine keratinocytes Cell Growth Differ 2000 11 447 454 10965849

[B24] Delerive P Furman C Teissier E Fruchart JC Duriez P Staels B Oxidized phospholipids activate PPARα in a phospholipase A2-dependent manner FEBS Lett 2000 471 34 38 10.1016/S0014-5793(00)01364-8 10760508

[B25] Garelnabi M Selvarajan K Litvinov D Santanam N Parthasarathy S Dietary oxidized linoleic acid lowers triglycerides via APOA5/APOClll dependent mechanisms Atherosclerosis 2008 199 304 309 10.1016/j.atherosclerosis.2007.12.026 18243209 PMC2562931

[B26] Chinetti G Fruchart JC Staels B Peroxisome proliferator-activated receptors: new targets for the pharmacological modulation of macrophage gene expression and function Curr Opin Lipidol 2003 14 459 468 10.1097/00041433-200310000-00006 14501584

[B27] Chinetti G Lestavel S Fruchart JC Clavey V Staels B Peroxisome proliferator-activated receptor α reduces cholesterol esterification in macrophages Circ Res 2003 92 212 217 10.1161/01.RES.0000053386.46813.E9 12574149

[B28] Chawla A Boisvert WA Lee CH Laffitte BA Barak Y Joseph SB Liao D Nagy L Edwards PA Curtiss LK Evans RM Tontonoz P PPARγ-LXR-ABCA1 Pathway in macrophages is involved in cholesterol efflux and atherogenesis Mol Cell 2001 7 161 171 10.1016/S1097-2765(01)00164-2 11172721

[B29] Lusis AJ Atherosclerosis Nature 2000 407 233 241 10.1038/35025203 11001066 PMC2826222

[B30] Wang R Kern JT Goodfriend TL Ball DL Luesch H Activation of the antioxidant response element by specific oxidized metabolites of linoleic acid Prostaglandins Leukot Essent Fatty Acids 2009 81 53 59 10.1016/j.plefa.2009.04.008 19481916 PMC2756043

[B31] Willker W Leibfritz D Lipid oxidation in blood plasma of patients with neurological disorders Brain Res Bull 2000 53 437 443 10.1016/S0361-9230(00)00375-0 11137001

[B32] Spiteller P Spiteller G 9-Hydroxy-10-12-octadecadienoic acid (9-HODE) and 13-hydroxy-9,11-octadecadienoic acid (13-HODE): excellent markers for lipid peroxidation Chem Phys Lipids 1997 89 131 139 10.1016/S0009-3084(97)00070-4

[B33] Ringseis R König B Leuner B Schubert S Nass N Stangl G Eder K LDL receptor gene transcription is selectively induced by t10c12-CLA but not by c9t11-CLA in the human hepatoma cell line HepG2 Biochim Biophys Acta 2006 1761 1235 1243 16982210 10.1016/j.bbalip.2006.08.007

[B34] Bull AW Steffensen KR Leers J Rafter JJ Activation of PPARγ in colon tumor cell lines by oxidized metabolites of linoleic acid, endogenous ligands for PPAR γ Carcinogenesis 2003 24;1717 1722 10.1093/carcin/bgg131 12949056

[B35] Nagy L Tontonoz P Alvarez JG Chen H Evans RM Oxidized LDL regulates macrophage gene expression through ligand activation of PPARγ Cell 1998 93 229 240 10.1016/S0092-8674(00)81574-3 9568715

[B36] Lorenzi I von Eckardstein A Cavelier C Radosavljevic S Rohrer L Apolipoprotein A-I but not high-density lipoproteins are internalised by RAW macrophages: roles of ATP-binding cassette transporter A1 and scavenger receptor BI J Mol Med 2008 86 171 183 10.1007/s00109-007-0267-1 17906976

[B37] Rong R Ramachandran S Penumetcha M Khan N Parthasarathy S Dietary oxidized fatty acids may enhance intestinal apolipoprotein A-I production J Lipid Res 2002 43 557 564 11907138

[B38] Eder K Keller U Brandsch C Effects of a dietary oxidized fat on cholesterol in plasma and lipoproteins and the susceptibility of low-density lipoproteins to lipid peroxidation in guinea pigs fed diets with different concentrations of vitamins E and C Int J Vitam Nutr Res 2004 74 11 20 10.1024/0300-9831.74.1.11 15060896

[B39] Ringseis R Piwek N Eder K Oxidized fat induces oxidative stress but has no effect on NF-κB-mediated proinflammatory gene transcription in porcine intestinal epithelial cells Inflamm Res 2007 56 118 125 10.1007/s00011-006-6122-y 17406809

[B40] Tancevski I Wehinger A Schgoer W Eller P Cuzzocrea S Foeger B Patsch JR Ritsch A Aspirin regulates expression and function of scavenger receptor-BI in macrophages: studies in primary human macrophages and in mice FASEB J 2006 20 1328 1335 10.1096/fj.05-5368com 16816107

[B41] Toh SA Millar JS Billheimer J Fuki I Naik SU Macphee C Walker M Rader DJ PPARγ activation redirects macrophage cholesterol from fecal excretion to adipose tissue uptake in mice via SR-BI Biochem Pharmacol 2011 81 934 941 10.1016/j.bcp.2011.01.012 21291868 PMC3315103

[B42] Malerød L Juvet LK Hanssen-Bauer A Eskild W Berg T Oxysterol-activated LXRα/RXR induces hSR-BI-promoter activity in hepatoma cells and preadipocytes Biochem Biophys Res Commun 2002 299 916 923 10.1016/S0006-291X(02)02760-2 12470667

[B43] Laffitte BA Joseph SB Walczak R Pei L Wilpitz DC Collins JL Tontonoz P Autoregulation of the human liver × receptor alpha promoter Mol Cell Biol 2001 21 7558 7568 10.1128/MCB.21.22.7558-7568.2001 11604492 PMC99927

[B44] Krey G Braissant O L'Horset F Kalkhoven E Perroud M Parker MG Wahli W Fatty acids, eicosanoids, and hypolipidemic agents identified as ligands of peroxisome proliferator-activated receptors by coactivator-dependent receptor ligand assay Mol Endocrinol 1997 11 779 791 10.1210/me.11.6.779 9171241

[B45] Yu Y Correll PH Vanden Heuvel JP Conjugated linoleic acid decreases production of pro-inflammatory products in macrophages: evidence for a PPARγ-dependent mechanism Biochim Biophys Acta 2002 1581 89 99 12020636 10.1016/s1388-1981(02)00126-9

[B46] Ringseis R Wen G Saal D Eder K Conjugated linoleic acid isomers reduce cholesterol accumulation in acetylated LDL-induced mouse RAW264.7 macrophage-derived foam cells Lipids 2008 43 913 923 10.1007/s11745-008-3226-x 18769950

[B47] Weldon S Mitchell S Kelleher D Gibney MJ Roche HM Conjugated linoleic acid and atherosclerosis: no effect on molecular markers of cholesterol homeostasis in THP-1 macrophages Atherosclerosis 2004 174 261 273 10.1016/j.atherosclerosis.2004.02.007 15136056

[B48] Nagelin MH Srinivasan S Lee J Nadler JL Hedrick CC 12/15-Lipoxygenase activity increases the degradation of macrophage ATP-binding cassette transporter G1 Arterioscler Thromb Vasc Biol 2008 28 1811 1819 10.1161/ATVBAHA.108.167908 18635820 PMC2749732

[B49] Wang Y Oram JF Unsaturated fatty acids inhibit cholesterol efflux from macrophages by increasing degradation of ATP-binding cassette transporter A1 J Biol Chem 2002 277 5692 5697 10.1074/jbc.M109977200 11741998

[B50] Uehara Y Engel T Li Z Goepfert C Rust S Zhou X Langer C Schachtrup C Wiekowski J Lorkowski S Assmann G von Eckardstein A Polyunsaturated fatty acids and acetoacetate downregulate the expression of the ATP-binding cassette transporter A1 Diabetes 2002 51 2922 2928 10.2337/diabetes.51.10.2922 12351428

[B51] Uehara Y Miura S von Eckardstein A Abe S Fujii A Matsuo Y Rust S Lorkowski S Assmann G Yamada T Saku K Unsaturated fatty acids suppress the expression of the ATP-binding cassette transporter G1 (ABCG1) and ABCA1 genes via an LXR/RXR responsive element Atherosclerosis 2007 191 11 21 10.1016/j.atherosclerosis.2006.04.018 16730733

[B52] Ringseis R Eder K Fatty acids and signalling in endothelial cells Prostaglandins Leukot Essent Fatty Acids 2010 82 189 198 10.1016/j.plefa.2010.02.022 20207525

[B53] Chinetti-Gbaguidi G Rigamonti E Helin L Mutka AL Lepore M Fruchart JC Clavey V Ikonen E Lestavel S Staels B Peroxisome proliferator-activated receptor α controls cellular cholesterol trafficking in macrophages J Lipid Res 2005 46 2717 2725 10.1194/jlr.M500326-JLR200 16162941

[B54] Carstea ED Morris JA Coleman KG Loftus SK Zhang D Cummings C Gu J Rosenfeld MA Pavan WJ Krizman DB Nagle J Polymeropoulos MH Sturley SL Ioannou YA Higgins ME Comly M Cooney A Brown A Kaneski CR Blanchette-Mackie EJ Dwyer NK Neufeld EB Chang TY Liscum L Strauss JF Ohno K Zeigler M Carmi R Sokol J Markie D O'Neill RR van Diggelen OP Elleder M Patterson MC Brady RO Vanier MT Pentchev PG Tagle DA Niemann-Pick C1 disease gene: homology to mediators of cholesterol homeostasis Science 1997 277 228 231 10.1126/science.277.5323.228 9211849

[B55] Hu Q Zhang XJ Liu CX Wang XP Zhang Y PPARγ1-induced caveolin-1 enhances cholesterol efflux and attenuates atherosclerosis in apolipoprotein E-deficient mice J Vasc Res 2010 47 69 79 10.1159/000235927 19729954

[B56] Schild RL Schaiff WT Carlson MG Cronbach EJ Nelson DM Sadovsky Y The activity of PPARγ in primary human trophoblasts is enhanced by oxidized lipids J Clin Endocrinol Metab 2002 87 1105 1110 10.1210/jc.87.3.1105 11889173

[B57] Aperlo C Pognonec P Saladin R Auwerx J Boulukos KE cDNA cloning and characterization of the transcriptional activities of the hamster peroxisome proliferator-activated receptor haPPARγ Gene 1995 162 297 302 10.1016/0378-1119(95)00196-D 7557447

